# Delivery of Alginate Scaffold Releasing Two Trophic Factors for Spinal Cord Injury Repair

**DOI:** 10.1038/srep13702

**Published:** 2015-09-08

**Authors:** I. Grulova, L. Slovinska, J. Blaško, S. Devaux, M. Wisztorski, M. Salzet, I. Fournier, O. Kryukov, S. Cohen, D. Cizkova

**Affiliations:** 1 Institute of Neurobiology, Center of Excellence for Brain Research, Department of Regenerative Medicine and Stem Cell Therapy, Slovak Academy of Sciences, Soltesovej 4-6, 040 01 Kosice, Slovakia; 2 Laboratoire PRISM: Protéomique, Réponse Inflammatoire, Spectrométrie de Masse, INSERM U1192, Bât SN3, 1er étage, Université de Lille 1, F-59655 Villeneuve d’Ascq, France; 3 The Center of Regenerative Medicine and Stem Cell Research and The Avram and Stella Goldstein-Goren Department of Biotechnology Engineering, Ben-Gurion University of the Negev, Beer Sheva, Israel

## Abstract

Spinal cord injury (SCI) has been implicated in neural cell loss and consequently functional motor and sensory impairment. In this study, we propose an alginate -based neurobridge enriched with/without trophic growth factors (GFs) that can be utilized as a therapeutic approach for spinal cord repair. The bioavailability of key GFs, such as Epidermal Growth factor (EGF) and basic Fibroblast Growth Factor (bFGF) released from injected alginate biomaterial to the central lesion site significantly enhanced the sparing of spinal cord tissue and increased the number of surviving neurons (choline acetyltransferase positive motoneurons) and sensory fibres. In addition, we document enhanced outgrowth of corticospinal tract axons and presence of blood vessels at the central lesion. Tissue proteomics was performed at 3, 7 and 10 days after SCI in rats indicated the presence of anti-inflammatory factors in segments above the central lesion site, whereas in segments below, neurite outgrowth factors, inflammatory cytokines and chondroitin sulfate proteoglycan of the lectican protein family were overexpressed. Collectively, based on our data, we confirm that functional recovery was significantly improved in SCI groups receiving alginate scaffold with affinity-bound growth factors (ALG +GFs), compared to SCI animals without biomaterial treatment.

Spinal cord injury (SCI) involves a multifactorial process that initiates pathological cellular and molecular responses resulting in limited spontaneous axonal regeneration^1^. Clinical symptoms following trauma can vary in severity, but usually lead to complete paralysis and spasticity^1^,^2^,^3^,^4^. The development of a safe and efficient treatment for spinal cord injuries is greatly complicated by the existence of a highly complex injury environment. Over the past decades various strategies have been proposed including inflammatory processes and suppression of edema^5^,^6^, promotion of axonal regeneration through the decrease of inhibitory molecules^7^,^8^,^9^, transplantation of stem cells to replace lost tissue, or enhancement of endogenous repair with trophic factor support and rehabilitative training^10^,^11^,^12^. All these strategies were developed to target specific pathological players during secondary damage, whereas nowadays a combinatorial approach integrating biomaterial scaffolds, cell transplantation and molecule delivery seems to be more promising for regeneration and functional recovery^13^,^14^,^15^,^16^.

An attractive strategy for repairing injured spinal cord is to incorporate multiple neurotrophic factors in biodegradable and biocompatible microspheres, or injectable matrices that allow controlled, sustained and localized delivery of those factors^17^,^18^. The alginate scaffold is a suitable biomaterial construct providing a cellular mechanical framework of polysaccharide chains that gels by ionic cross linking after mixing aqueous alginate solution with divalent cations such as Ca^2+^^19^. Natural substrate isolated from the wall of brown seaweed represents a non-toxic/non-inflammatory, highly porous scaffold with relatively low cost^20^. Alginate hydrogel has been widely used for drug or cell delivery as an injectable vehicle capable of filling cavities in the injured spinal cord^21^,^22^,^23^, and of providing the substrate for axon attachment and re-growth^15^,^20^,^24^.

Along these lines, we have recently reported that an affinity-binding alginate scaffold which sustains the release and presentation of both epidermal growth factor (EGF) and fibroblast growth factor-2 (bFGF) is capable of supporting the viability, expansion and lineage differentiation of neural progenitor cells (NPCs) *in vitro*^25^. Following these findings, our next goal was to test this scaffold with its affinity-bound growth factors (GFs) for treatment of spinal cord compression in rats. Although increasing evidence demonstrates that the adult spinal cord harbours a population of multipotent neural precursor cells (NPCs), which is further increased by injury, their ability to replace lost neuroglia populations has been shown to be insufficient. Optimizing the SCI environment, therefore, by enhancing the presence and longevity of EGF and bFGF may provide an appropriate supportive microenvironment for the survival, and integration of endogenous NPCs into functional neural circuitry *in situ*^14^,^26^,^27^,^28^. The GFs selected for this study share important roles in regulating NPC proliferation and differentiation: bFGF is a known mitogen for stem cell self-renewal, while EGF induces both proliferation and differentiation in many mammalian stem cells^29^ including NPCs. Furthermore, both GFs accelerate neovascularisation, necessary for supporting and rebuilding the damaged tissue.

In the present study, we performed biomaterial treatment at 7 days after SCI followed by tissue microproteomics and immunocytochemistry. Our microproteomic data based on high-resolution (HR) MS/MS shot-gun procedures and statistical analyses^30^ indicated that the caudal region 7–10 days post SCI compared to that of 3 days post SCI is able to initiate neurogenesis if trophic and inflammatory inhibitors factors are present on-site. We therefore addressed the therapeutic efficacy of the affinity-binding alginate scaffold as described herein in terms of functional recovery and nerve tissue repair. Specifically, we investigated its influence on: (i) tissue sparing *via* reduction of the central cavity and enhanced survival of neuronal populations, ii) neurite outgrowth, iii) angiogenesis, iv) response of astrocytes and microglia involved in inflammation and scarring, and v) functional recovery of sensory-motor pathways during a period of 49 days after SCI in rats.

## Materials and Methods

### Experimental groups

Male Wistar albino rats weighing 290–320 g were divided into 5 groups: 1) sham-operated SCI group (n = 6), 2) sham-operated and SCI rats (ALG+ALG+GFs) after biotinylated dextran amines (BDA) tracing (n = 10), 3) SCI group receiving saline injection (SCI+SAL) (n = 8), 4) SCI group receiving an injection of alginate scaffold/lacking growths factors (SCI+ALG) (n = 8), and 5) SCI group receiving an injection of alginate scaffold with affinity-bound EGF and bFGF (SCI+ALG+GFs) (n = 8). During the survival, rats were behaviourally tested and after 49 day post-injury, all groups were sacrificed and spinal cord tissue was processed for immunohistochemistry and tracing analysis. A set of 12 animals subjected to SCI at 3, 7, 10 days (n = 4 for each time point) was used for proteomic analyses.

### Animals

The study was performed with the approval and according to the guidelines of the Institutional Animal Care and Use Committee of the Slovak Academy of Sciences and with the European Communities Council Directive (2010/63/EU) regarding the use of animals in Research, Slovak Law for Animal Protection No. 377/2012 and 436/2012. In present study we used a total of 40 rats, and 34 survived.

### Spinal cord injury

The SCI was induced using the modified balloon-compression technique according to our previous study^31^. Briefly, 2-French Fogarty catheter was inserted epidurally at Th8-9 level and the balloon was inflated with 12.5 μl of saline for 5 min. After compression of spinal cord tissue, catheter was deflated and removed from epidural space. In the sham group (n = 4), the catheter was inserted at the same level of spinal cord, but balloon was not inflated and no lesion was performed. Manual bladder expression was required for 7–14 days after the injury until the bladder reflex was established. No antibiotic treatment was used.

### Tissue protein extraction

Fresh frozen spinal cord collected after 3, 7 and 10 days after lesion and controls were embedded (n = 4, each group) on optimal cutting temperature polymer before sectioned using a cryomicrotome (Leica Microsystems, Nantere, France) and subjecting to trypsin digestion. Spinal cord tissue sections (20 μm thick) were mounted on a parafilm covered glass slide and the tissue was microdissected manually using a binocular. The pieces were extracted by incubating in 20 μL of 50 mM bicarbonate buffer containing 50 mM dithiothreitol and 1% SDS at 55 °C for 15 min. The extracts were then loaded on 12% polyacrylamide gel and separated at 70 V for 15 min and then 120 V until the dye front reaches the other end of the gel. After migration, the gel was incubated in the gel fixative solution for 30 min and stained with colloidal Coomassie brilliant blue overnight. The stain was removed by washing the gel four times with distilled deionized water^30^.

### In gel digestion

The gel was cut into ten pieces. Pieces were washed with 300 μL of distilled deionized water for 15 min, 300 μL of ACN for 15 min and 300 μL of NH_4_HCO_3_ (100 mM; pH8) for 15 min followed by incubation of 300 μL of NH_4_HCO_3_/acetonitrile (ACN) (1:1, v/v) for 15 min and 300 μL of ACN for 5 min. Band pieces were dried in a Speedvac for 5 min. The reduction of cystine residues was made with 50 μL of 10 mM of DTT in NH_4_HCO_3_ 100 mM (pH8). Pieces were incubated at 56 °C for 1 hour. Alkylation of cystine was made with 50 μL of of iodo acetamide IAA (50 mM) in NH_4_HCO_3_ (100 mM; pH8). Pieces were incubated at room temperature in the dark for 30 min. Band pieces were washed a second time with 300 μL of NH_4_HCO_3_ 100 mM (pH8) for 15 min, then with a mix of 300 μL of NH_4_HCO_3_/ACN (1:1, v/v) for 15 min and 300 μL of ACN for 5 min. Band pieces were dried in a Speedvac for 5 min. A digestion of band pieces was made with trypsin (12.5 μg/mL) in NH_4_HCO_3_ 20 mM (pH8), enough to cover pieces. Pieces were incubated at 37 °C overnight. Peptides were extracted on shaking platform with 50 μL of formic acid (FA) 1% two times for 20 min, then 150 μL of ACN for 10 min. The supernatant was transferred in new tube and dried with Speedvac. Samples were resuspended in 20 μL of 0.1% trifluoro acetic acid (TFA) and then desalted with Ziptip C18 and eluted with 10 μL of ACN/0.1% TFA (8:2, v/v). Samples were dried in a Speedvac and resuspended in 15 μL of ACN/0.1% FA (2:98, v/v).

### NanoLiquid Chromatography –High Resolution-MS/MS (NanoLC-HR- MS/MS)

Samples were separated by online reversed-phase chromatography using a Thermo Scientific Proxeon Easy-nLC system equipped with a Proxeon trap column (100 μm ID × 2 cm, Thermo Scientific) and a C18 packed-tip column (100 μm ID × 10 cm, Nikkyo Technos Co. Ltd). Peptides were separated using an increasing amount of acetonitrile (5%–30% over 120 minutes) at a flow rate of 300 nL/min. The LC eluent was electrosprayed directly from the analytical column and a voltage of 1.6 kV was applied via the liquid junction of the nanospray source. The chromatography system was coupled to a Thermo Scientific LTQ-Orbitrap XL mass spectrometer. The LTQ-Orbitrap XL instrument was set to acquire top 20 MS/MS in data-dependent mode. The survey scans were taken at 70,000 full width at half maximum (FWHM) (at m/z 400) resolving power in positive mode and using a target of 3E6 and default charge state of 2. Unassigned and +1 charge states were rejected, and dynamic exclusion was enabled for 20 s. The scan range was set to 300–1600 m/z. For the MS/MS, 1 microscan was obtained at 17,500 FWHM and isolation window of 4.0 m/z, using a scan range between 200–2000 m/z 30,32.

### Mass Spectra Data Analysis

Tandem mass spectra were processed with Thermo Scientific Proteome Discoverer software version 1.3. Resultant spectra were searched against the Swiss-Prot® *Rattus norvergicus* database (version January 2012) using the SEQUEST® algorithm. The search was performed choosing trypsin as the enzyme with two missed cleavages allowed. Precursor mass tolerance was 10 ppm, and fragment mass tolerance was 0.5 Da. N-terminal acetylation, methionine oxidation and arginine deamination were set as variable modifications. Peptide validation was performed with the Percolator algorithm. Peptides were filtered based on a q-Value below 0.01, which corresponds to a false discovery rate (FDR) of 1%. All the MS data were processed with MaxQuant^33^ (version 1.5.1.2) using Andromeda^34^ search engine. Proteins were identified by searching MS and MS/MS data against Decoy version of the complete proteome for Rattus norvegicus of the UniProt database [UniProt Consortium. Reorganizing the protein space at the Universal Protein Resource (UniProt). Nucleic Acids Res. 2012, 40 (Database issue), D71−5.] (Release June 2014, 33675 entries) combined with 262 commonly detected contaminants. Trypsin specificity was used for digestion mode, with N-terminal acetylation and methionine oxidation selected as variable, carbarmidomethylation of cysteines was set as a fixed modification and we allow up to two missed cleavages. For MS spectra an initial mass accuracy of 6 ppm was selected and the MS/MS tolerance was set to 0.5 Th for CID data. For identification, the FDR at the peptide spectrum matches (PSM) and protein level was set to 0.01. Relative, label-free quantification of proteins was done using the MaxLFQ algorithm^35^ integrated into MaxQuant with the default parameters.

The data sets used for analysis are deposited at the ProteomeXchange Consortium^36^ (http://proteomecentral.proteomexchange.org) via the PRIDE partner repository^37^ with the dataset identifier.

Analysis of the proteins identified was done using Perseus software (http://www.perseus-framework.org/) (version 1.5.0.31). The file containing the information from identification was used and hits to the reverse database, proteins only identified with modified peptides and potential contaminants were removed. Then the LFQ intensity were logarithmized (log2(x)). A normalization was achieved using a Z-score with a matrix access by rows. Data coming from control samples were average as the one coming from the lesion part. Six conditions were then analyzed: control (ctrl), lesion part (lesion), segment R1 or C1 seven days (respectively r1_7D and C1_7D) or ten days (r1_10D and C1_10D) after lesion. Only proteins presenting a valid value of LFQ intensity for these six conditions were used for statistical analysis. A Hierarchical clustering was first performed using a Pearson correlation for distance calculation and average option for linkage in row and column trees using a maximum of 300 clusters. For visualization of the variation of proteins expression depending to the segment/time parameter, the profile plot tool was used with a reference profile and an automatic selection of the 10 or 15 correlated profiles.

### Preparation of alginate scaffold with affinity-bound factors

Fabrication of the scaffold with the affinity-bound dual growth factors (ALG+GFs) involved preparing bioconjugates of bFGF and EGF with alginate-sulfate and then mixing both bioconjugate solutions with the solution of a partially calcium-cross-linked alginate. The bioconjugates were prepared by mixing bFGF or EGF with alginate-sulfate solution (1%, w/v) and incubating for 1.5 h at 37 °C, to allow equilibrium binding. The partially calcium-cross-linked alginate solution was prepared as previously described^38^. Briefly, stock solutions of sodium alginate (VLVG, 30–50 kDa, >65% guluronic acid content, NovaMatrix FMC Biopolymers, Drammen, Norway) and D-gluconic acid/hemi calcium salt were prepared by dissolving the materials in DDW and stirring at room temperature. Each solution was filtered separately through a sterile 0.2 μm filter membrane into a sterile container in a laminar flow cabinet. Equal volumes from each stock solution (2.08% and 0.68% (w/v) for VLVG alginate and D-gluconic acid, respectively) were combined by extensive homogenization for several minutes to facilitate homogenous distribution of the calcium ions and cross linking of alginate chains. Finally, the bFGF and EGF alginate-sulfate bioconjugates were mixed with the partially cross-linked alginate to yield an bFGF/EGF-containing, affinity-binding alginate scaffold (0.1% alginate-sulfate, 0.9% alginate, 0.3% D-gluconic acid, w/v) (ALG+GFs). For the control system- lacking GFs, the scaffold was prepared with no affinity-bound factors (ALG).

### Intraspinal delivery of alginate scaffold

Seven days after SCI, animals were anesthetized with 1.5–2% halothane and partial laminectomy at Th6-12 level was performed. Using a 50-μl Hamilton syringe (30G needle, Cole Parmer, Anjou, Quebec) connected to UltraMicroPump III with Micro4 Controller, 4-Channel (World Precious Instruments, Inc., Sarasota FL) and stereotactic device, 4 intraspinal injections/per animal were applied at the lesion site that showed discreet signs of haemorrhage and slight atrophy. In most cases the lesion cavity was apparent through the dorsal site of spinal cord. Bilateral delivery of i) saline, ii) ALG, or iii) ALG+GFs (2 injections of 2 μl/per injection/on left and 2 injections of 2 μl/per injection/on right side with delivery rate of 0.5 μl/min, loaded with 200 ng/ml of each GF) was performed. Based on our *in vitro* study results, an affinity-binding alginate scaffold loaded with 200 ng of bFGF/EGF confirmed long term release of GFs^25^. Each delivery was positioned 1 mm from the spinal cord midline and injected at the depth of 1.8–2 mm from the pial surface of the spinal cord. The distance between injections was 1 mm, avoiding vessels. After injecting the dose of alginate scaffold, the needle was maintained in the tissue for an additional 30 seconds. No antibiotic treatment was performed during animal’s survival.

### Anterograde biotinilated dextran amine (BDA) motor corticospinal tract (CST) axon tracing

Sham rats (n = 4) and SCI rats (ALG (n = 3), and ALG+GFs (n = 3)) at 3 weeks post-injury were anesthetized with 2% halothane and placed in a stereotaxic device. The halothane level was maintained at 2–3% throughout the surgery. An incision was made to expose the skull and to identify the bregma and lambda landmarks. Rats received injections of 10% solution of BDA (biotinilated dextran amine 10,000 MW; Molecular Probes, Eugene, OR) in sterile 10 mM sodium phosphate buffer, pH 7.4, injected via glass micropipettes (inner tip diameter of 60–80 μm) using a controlled pressure device (PicoPump; World Precision Instruments). The injection site was positioned into right and left motor cortex performed at anatomical coordinates: 1.0 mm lateral to bregma, 1.5 mm anterior/posterior to the bregma and 1.5 mm deep to the cortical surface from the pial surface of the brain based on the Stereotaxic Coordinates (Paxinos and Franklin, 2001) (Supplementary Figure 1). A total 8 injections with approximately 0.5 μl of BDA was injected at each of the four sites at a rate of 80 nl/min during 6–7 min/per injection. The micropipette remained in place for 3 minutes following each injection. After the delivery was completed, the skin overlying the skull was sutured and rats returned to their cages.

### Behavioral Testing

#### BBB scoring

Animals were behaviourally tested for 5 min using BBB open-field locomotor test^39^ after SCI at day 1, 3, 5, 7, and then in weekly intervals. Each rat was tested for 5 min by two blinded examiners. BBB test measuring locomotor outcome (hindlimb activity, body position, trunk stability, tail position and walking paw placement) of rats by BBB rating scale ranges from 0 - no observable hindlimbs movements to a maximum 21 - plantar stepping, coordination and trunk stability like healthy rats.

#### Cold allodynia

Cold sensitivity of the hindpaws to the acetone was quantified by foot withdrawal frequency. All animals were tested at day 3 post-injury and then in weekly intervals at day 16, 25, 32 and 49 after SCI. Before testing, the rats were left to acclimatize inside acrylic-plastic cages during the 10–15 min. A drop of acetone (50–100 μl) was applied to the left and right hindpaws using a plastic syringe, 5 times, with at least 5 min recovery between administrations. The number of brisk foot withdrawals or flinching were considered to be positive behaviour. Data are presented as mean response duration (in seconds). Statistical differences between groups were determined with an unpaired Student’s t test.

The sequence of surgical and behavioural procedures performed in time are described in Supplementary Figure 2.

#### Tissue Processing and Immunohistochemistry

After a 49 day survival period, animals were deeply anesthetized by intraperitoneal thiopental injection (50 mg/kg) and perfused transcardially with 500 ml saline, followed by 500 ml of 4% paraformaldehyde (PFA) in 0.1 M phosphate-buffered. Spinal cords were removed, postfixed in 4% PFA at 4 °C overnight, embedded in gelatin–egg albumin protein matrix (10% ovalbumin, 0.75% gelatine) polymerized by glutaraldehyde (albumin from chicken egg white, grade II, Sigma–Aldrich) subsequently fixed in 4% PFA, and cryoprotected with 30% sucrose in 0.1 M PB at 4 °C. Cryostat transversal and sagittal spinal cord sections (40 μm) were cut from rostral, central or caudal blocks (each 0.5 cm thick) (Supplementary Figure 3) and collected in 24-well plates with 0.1 M PBS containing 0.1% sodium aside. For immunohistochemistry, free floating sections (40 μm) were immersed in PBS (0.1 M; pH 7.4) containing 10% normal goat or normal rabbit serum (NGS, NRS), 0.2% Triton X-100 for 2 h at room temperature to block non-specific protein activity. This was followed by overnight incubation at 4 °C with primary antibodies: mouse mouse anti-neuronal nuclei antigen (NeuN; 1:500, Merck-Millipore), goat anti-Choline acetyltransferase (ChAT; 1:5000, Merck-Millipore), rabbit anti-calcitonine gene related protein (CGRP; 1:100, Merck-Millipore), rabbit anti- ionized calcium-binding adapter molecule 1 (Iba1; 1:500, Wako), mouse anti- glial fibrillary acidic protein (GFAP; 1:1000, Merck-Millipore) rabbit anti-vWF (1:200, Chemicon) and mouse anti-synaptophysin (SYN; 1:500, Merck-Millipore) for 24 h. Afterwards sections were washed in 0.1 M PBS and incubated with secondary fluorescent antibodies goat anti-mouse, goat anti-rabbit, rabbit anti-goat conjugated with Texas Red (Alexa Flour 594) and fluorescein isothiocyanate (FITC) (Alexa Flour 488) at room temperature for 2 hours. For general nuclear staining 4-6-diaminidino-2-phenylindol (DAPI) (1:200) was added to the final secondary antibody solutions. Finally, sections were mounted and coverslipped with Vectashield mounting medium (Vector Laboratories).

Serial sagittal sections from each animal were stained for BDA to determine labelled CST axons through the lesion site. Sections were washed in TBS (50 mM Tris/HCl, 150 mM NaCl, pH = 7.6) and subsequently incubated overnight at 4 °C with ABC kit (ABC Vectastain Ellite kit; Vector Laboratories) diluted in TBS. After rinsing 3 times for 10 minutes in TBS, sections were reacted with DAB (consisting of DAB substrate, DAB buffer and 0.6% NISO_4_) for 20 minutes. Sections were finally washed in TBS and coverslipped in Enthelan. Similarly serial sagittal sections (n = 5) were stained with Luxol Fast Blue to determine the length and cavity size area^40^. Schematic concept of dissected spinal cord segments consisting of rostral (Th5-7), central (Th8-9), caudal (Th10-12) segments processed for immunohistochemical analyses is included in (Supplementary Figure 3).

### Quantification analysis

Immunochemicaly stained sections were analyzed using Olympus BX-50 fluorescent microscope at 4x, 10x 20x and 40x magnifications, captured with digital camera HP Olympus and analyzed by Image J software according to the previous protocol^41^,^42^,^43^. Quantification of NeuN, ChAT, Iba1, CGRP, GFAP and SYN positive cells was performed on five transverse sections from rostral and caudal segments of each spinal cord treatment and from sham tissue. Similarly, for quantification of BDA tracing, GFAP, Iba1, vWF expression and cavity size in sagittal sections from lesion epicentre, five sections per each experimental animal were analyzed. Number of NeuN positive cells was evaluated through sample field of 200 μm × 200 μm, bilaterally positioned at Laminae I–IV (DH = area 1), IV–V (deep dorsal horn = area 2) and VIII–IX (ventral horn = area 3). Analysis of ChAT labelling was performed by using the identical field positioned in Laminae VIII–IX, bilaterally. Number of Iba1+ cells rostrally and caudally from injury epicenter was measured at the identical field in the Lamina VII gray matter (GM = area 1), lateral white matter (LWM = area 2) and ventral white matter (VWM = area 3) (Supplementary Figures 4 A,B). Quantification of immunofluorescence intensity (CGRP, Iba1, SYN) rostrally/caudally from lesion site and GFAP, Iba1 also at the site of central lesion) was performed by using ImageJ software. Captured digital images were transformed into monochrome 8-bit images and determined the mean grey level number of black and white pixels within the tissue (value 0–255, when 0 = black pixels, 255 = white pixels). The final result yields the mean ratio of black and white pixels expressed by the histogram. Length of BDA were equally evaluated by Image J and expressed in mm. Morphometric analyses of cavity size were performed on five 1.6 cm sagittal sections from the lesion site of each experimental and sham tissue. Modified Luxol Fast Blue labelling was performed to evaluate cavity area in spinal cord sections^40^. Mean number of cavitation of experimental groups was expressed by mm relative to Sham spinal cord, which was without cavitation’s and represent zero (no cavity).

### Data and statistical analysis

Obtained data from tissue analyses and behavioural testing were reported as mean ± SEM. Mean values among different experimental groups were statistically compared by one-way ANOVA and Tukey’s post hock tests using Graph pad PRISM software. Values of P < 0.05 were considered statistically significant (*P value of < 0.05, **P value of < 0.01, ***P < 0.001).

## Results

### Spatiotemporal proteomic study of spinal cord tissue after injury

**Figure 1:**
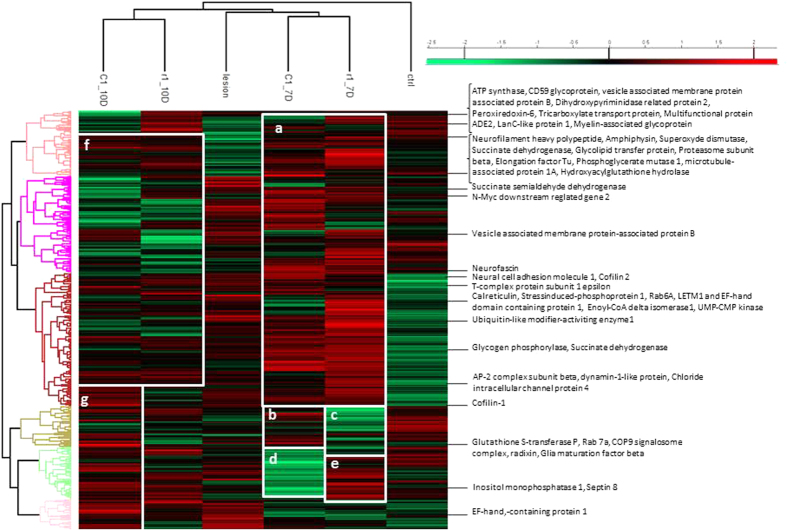
Heat Map of proteins from control, lesion, R1 (rostral segment adjacent to central lesion) at 7 and 10 days (7D and 10D), and C1 (caudal segment adjacent to central lesion) at 7 and 10 days. Green-subexpression, red-overexpression. (**a**) Protein cluster overexpressed at 7 days compared to 10 days. (**b**) Protein cluster overexpressed in C1 at 7 days compared to R1 at 7D. (**c**) Protein cluster subexpressed in R1 at 7D compared to C1 at 7D. (**d**) Protein cluster subexpressed in C1 at 7D compared to R1 at 7D. (**e**) Protein cluster overexpressed in R1 at 7D compared to C1 at 7D. (**f**) Protein cluster subexpressed at 10D compared to 7D. (**g**) Protein cluster overexpressed at 10D in C1.

Spinal cord tissues, the rostral, lesion and caudal segments, were collected at 7 and 10 days after lesion and were subjected to tissue microproteomic, MaxQuant proteins analyses followed by Perseus allowed to statistically validate the identification and performed clustering. Figure 1 clearly shows that the control tissue is on a separate branch from lesion, rostral and caudal segments. The time course study reflected that 7 and 10 days are separated from each other. Comparison of the data obtained at 7 days between rostral and caudal segments clearly shows a common cluster of over-expressed groups of proteins (Fig. 1a) and four different clusters between the rostral and caudal of over- or sub-expressed protein groups (Fig. 1b–e). Proteins overexpressed at 7 days (Fig. 1a) are sub-expressed at 10 days (Fig. 1f). Only a cluster of proteins is differentially overexpressed at the caudal level (Fig. 1g). Specific proteins overexpressed in lesion, rostral and caudal segments are presented in Table 1. Radixin, Neural cell adhesion molecule, COP9 signalosome complex (CSN); Cofilin 2; AP-2, dynamin-like, Rab-7a, GST_P are specific proteins that are overexpressed only in caudal regions at 7 and 10 days. In rostral segment 7 and 10 days after SCI, amphiphysin, hydroxyacyl glutathione hydrolase, inositol monophosphatase, Neurofilament heavy polypeptide, Glycogen phosphorylase, Superoxide dismutase [Cu-Zn], phosphoglycerate mutase 1, septin 8, microtubule-associated protein 1A, CD59 glycoprotein, LETM1 and EF-hand domain-containing protein 1, Elongation factor Tu, Succinate dehydrogenase [ubiquinone] flavoprotein subunit, Vesicle-associated membrane protein-associated protein B are the ones specifically overexpressed.

### Locomotor function recovery

**Figure 2:**
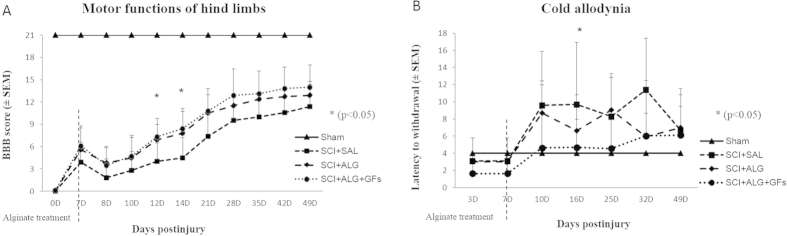
Functional recovery of hindlimb motor (**A**) and sensory functions (**B**) following SCI in Sham, SCI+SAL, SCI+ALG and SCI+ALG+GFs experimental groups. (**A**) *P < 0.05 indicates significant differences among the experimental groups.

During the initial days post-injury, the compression caused hindlimb paralysis with slight movement in one or two joints in all experimental groups. On following days, the animals in the SCI+ALG and SCI+ALG+GFs biomaterial treatment groups showed a similar gradual recovery of hindlimb locomotion up to 21D after SCI; greater than the recovery in the SCI+SAL group, where only limited recovery of motor function was noted. The significant locomotor improvement (*p < 0.05) between SCI+SAL and SCI+ALG/SCI+ALG+GFs was detected at 12 and 14 days post-injury with the final BBB scores 7.3 ± 2.5/12D, 8.4 ± 2.7/14D (SCI+ALG+GFs), 6.9 ± 2.1/12D, 7.8 ± 2.9/14D (SCI+ALG) and 4 ± 2.4/12D, 4.5 ± 2.714D (SCI+SAL) (Fig. 2A). The BBB score of hindlimb motor function gradually increased in all experimental groups with the survival (21, 28, 35, 42 and 49 days post-surgery), however the highest scores were observed in SCI+ALG+GFs (10.8 ± 3, 12.9 ± 3.6, 13.1 ± 1.2, 13.8 ± 3.1, 14.0 ± 3), which closely correlated with SCI+ALG (10.5 ± 2.5, 11.5 ± 1.4, 12.4 ± 0.9, 12.7 ± 0.9, 12.9 ± 1.9) but the increase was less prominent. The SCI+SAL group following initial gradual motor function improvement at 8 to 28 days, showed only limited recovery during further survival (7.4 ± 3.6/21D, 9.5 ± 3.6/28D, 10 ± 3.1/35D, 10.6 ± 2.9/42D, 11.4 ± 3/49D) (Fig. 2A).

### Cold allodynia

Increased sensitivity to normally non-painful cold stimulus is a characteristic feature of clinical neuropathic pain states. In our experiments, spinal cord injury result into adverse pain behaviour - development of cold allodynia with positive responses to cold stimulus as paw withdrawal, licking, lifting and shaking of the hindpaw. Increased sensitivity to the acetone application developed mainly at 10D post-injury in the saline (9.6 ± 6.2/SCI+SAL) and alginate (8.7 ± 7.8/SCI+ALG) treated groups. Low increase in sensitivity to a normally innocuous stimulus was observed in SCI+ALG+GFs during entire survival (4.6 ± 3.1/10D, 4.7 ± 3.8/16D, 4.6 ± 2.1/25D, 6 ± 2.7/32D, 6.1 ± 3.2/49D), compared to sham (4 ± 1.8/10, 16, 25, 32, 49D). Frequent and more severely aversive responses to the acetone stimulus retained in SCI+ALG group at 10D, 16D, 25D (8.7 ± 7.8/10D, 6.6 ± 3.3/16D, 9 ± 8.3/25D, 5.9 ± 2.6/32D) and SCI+SAL group until 49D post injury (9.6 ± 6.4/10D, 9.7 ± 7.2/16D, 8.3 ± 5/25D, 11.4 ± 6/32D, 6.7 ± 4.8/49D). Significant differences *p < 0.05) were observed among SCI+SAL (9.7 ± 7.2) and SCI+ALG+GFs (4.7 ± 3.8) groups at 16D post-injury (Fig. 2B).

### Cavity size

**Figure 3:**
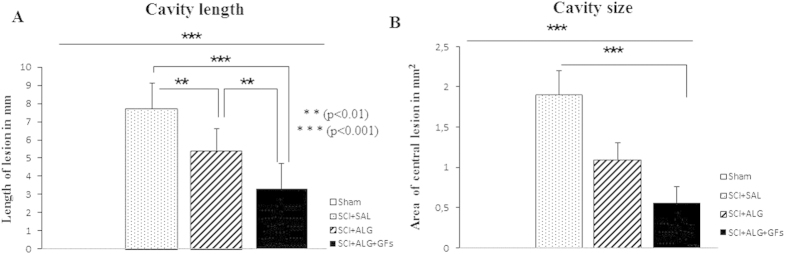
Morphometric analyses of cavity length (**A**) and size (**B**) in experimental groups showed significant reduction after intraspinal injection of biomaterial alginate (**P < 0.01, ***P < 0.001). Cavity size was expressed by mm in injured experimental groups (SCI+SAL, SCI+ALG and SCI+ALG+GFs) relative to Sham spinal cord without cavitations represent by zero.

During the first week post-injury, a severe inflammatory response occurs at the central lesion. The secondary damage processes lead to cell death and development of cavitations at the epicenter and along the rostrocaudal axis of the spinal cord^40^. In order to fill the cavity and create a permissive environment for regeneration, we administered the liquid form of alginate scaffold directly to the lesion cavity at 7D post injury. Histological assessment of spinal cord sections stained with Luxol Fast Blue revealed cavity area reduction in SCI+ALG+GFs and SCI+ALG groups compared to SCI+SAL at 42 day post-implantation (Fig. 3). Quantitative stereological analyses of tissue fenestration in 1.6 cm segment revealed significant (**P < 0.01, ***P < 0.001) reduction of cavitation (analysing length and area of cavity) after application of ALG+GFs (cavity length/3.3 ± 1.5 mm/area/0.56 ± 0.2 mm^2^) and ALG (cavity length/5.4 ± 1.2 mm/area/1.13 ± 0.2 mm^2^) compared to the saline treatment (cavity length/7.7 ± 1.4 mm/area/1.96 ± 0.3 mm^2^) (Fig. 3).

### Quantification of NeuN

**Figure 4:**
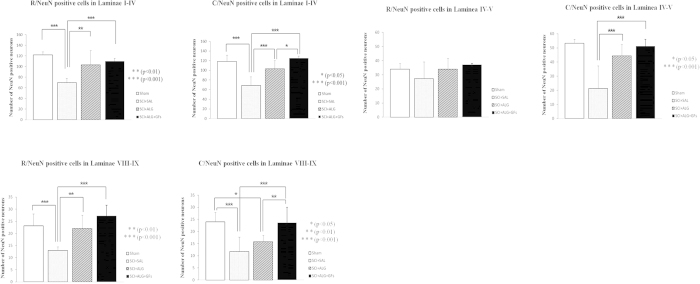
Stereological analyses of NeuN positive cells in Laminae I–IV, Laminae IV–V and Laminae VIII-IX after SCI and treatment. Number of NeuN labeled neurons in all studied areas increased after pure and enriched alginate administration compared to saline. Among the experimental groups we observed statistical differences (***P < 0.001, **P < 0.01, *P < 0.05).

Quantification analyses of representative transverse sections were processed bilaterally in dorsal (Laminae I–IV), ventral horns (Laminae VIII–IX) and Laminae IV–V, rostrally and caudally from the lesion site (Fig. 4). The higher number of NeuN-positive cells was documented rostrally from the injury site in all studied groups SCI+ALG+GFs, SCI+ALG and SCI+SAL. The most profound neuronal loss was observed in the SCI+SAL group (Rostral/Laminae I–IV 69.5 ± 8.1; Laminae VIII–IX 13 ± 1.5; Caudal/Laminae I–IV 68.8 ± 17.7; Laminae VIII–IX11.8 ± 5.8). The delivery of ALG or ALG+GFs promoted the survival of neuronal cells, resulting in a significant increase in number of NeuN-positive cells SCI+ALG: Rostral/Laminae I–IV 103.1 ± 26.6; Laminae VIII–IX 22 ± 5.5; Caudal/Laminae I–IV 103.3 ± 16.8; Laminae VIII–IX 15.8 ± 2,7; SCI+ALG+GFs: Rostral/Laminae I–IV 110 ± 5.3; Laminae VIII–IX 27.3 ± 4.4; Caudal/Laminae I–IV 125.5 ± 4.2; Laminae VIII–IX 23.6 ± 6.4). Moreover, the number of NeuN positive cells in the SCI+ALG+GFs group closely correlated with the NeuN numbers observed in Sham group (Rostral/Laminae I–IV 122 ± 5.2; Laminae VIII–IX 23.1 ± 5; Caudal/Laminae I–IV 120.7 ± 12.7; Laminae VIII–IX24 ± 3.8) (Fig. 4). The differences in NeuN positive profiles show a statistical significance between individual experimental groups: Sham, SCI+SAL, SCI+ALG, SCI+ALG+GFs ***P < 0.001, **P < 0.01, *P < 0.05.

### ChAT labeled motoneurons

**Figure 5:**
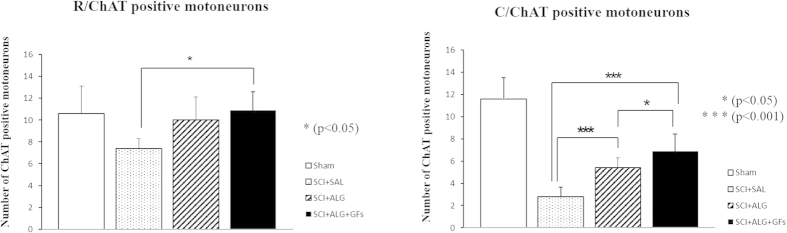
Quantification of ChAT labeled motoneurons in the Laminae VIII–IX. Marked depletion of motoneurons was observed caudally to the lesion site when compared with segment located rostrally. Significantly higher number of ChAT positive neurons was detected after delivery of enriched alginate in both studied segments (rostral, caudal) (***P < 0.001, *P < 0.05).

**Figure 6:**
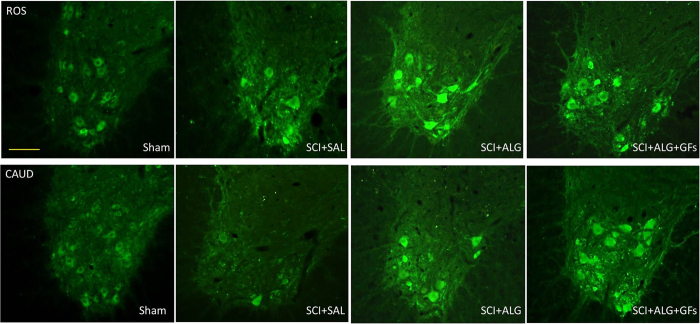
Representative transverse sections revealing ChAT positive motoneurons located rostrally and caudally to the lesion site following SCI in Sham, SCI+SAL, SCI+ALG and SCI+ALG+GFs experimental groups. Significant decrease of ChAT immunohistochemical staining was observed in ventral horns caudally to the lesion site (lower panel) following SCI and saline delivery. Injected alginate supports survival of ChAT positive motoneurons. Scale bar = 500 μm.

The average number of ChAT positive cells in the SCI+SAL, SCI+ALG and SCI+ALG+GFs groups was compared to confirm the hypothesis whether neuronal sparing has included motor neurons of ventral horns. Rostral to the lesion site, the number of spared ChAT+ neurons within the ventral horns significantly increased (*P < 0.05) following alginate biomaterial treatment (10 ± 2.1/SCI+ALG; 10.9 ± 1.7/SCI+ALG+GFs) when compared to the control saline group (7.4 ± 0.9) (Figs 5 and 6). Significant differences in sparing of motor neurons (*P < 0.05, ***P < 0.001) were also recorded among experimental groups caudal to the injury site, although the average number of positive cells had declined (6.9 ± 1.5/SCI+ALG+GFs, 5.4 ± 0.9/SCI+ALG, 2.8 ± 0.8/SCI+SAL, 11.9 ± 1.9/Sham) compared to spinal rostral part (Figs 5 and 6). Our results demonstrate that alginate biomaterial implantation resulted not only in common NeuN positive neurons sparing, but also in the specific sparing of endogenous ChAT+ motor neurons.

### Synaptic vesicles alterations

**Figure 7:**
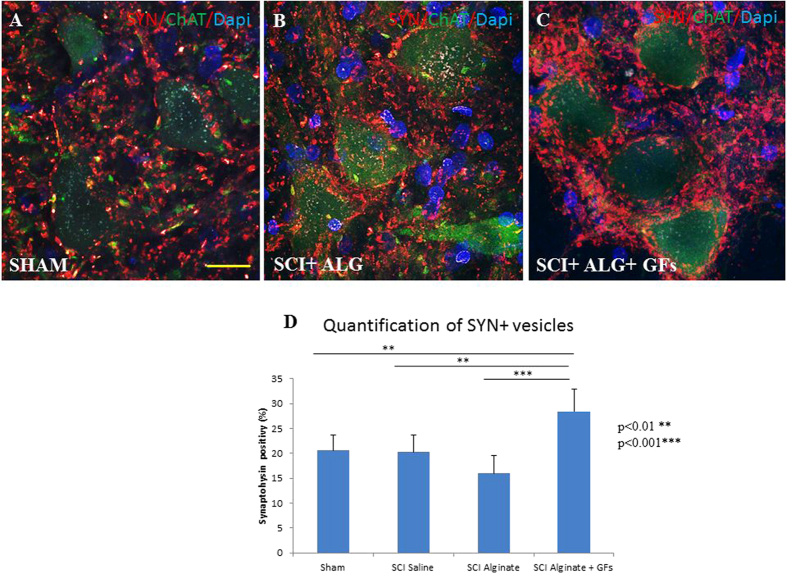
Distribution of synaptophysin positive vesicles (red) in the area of ChAT positive motoneurons (green) of the ventral horns. The density of vesicles after ALG+GFs treatment (**C**) is increased compared to both sham (**A**) and ALG without GFs group (**B**). (**D**) Quantification has revealed statistical significance only between ALG+GFs group and other experimental groups (SHAM, SCI+SAL, SCI+ALG). (**P < 0.01, ***P < 0.001). Scale bar = 50 μm.

In the spinal cord of Sham and both SCI-SAL and SCI-ALG groups of treated rats, synaptophysin immunoreactivity (SYN+IR) appeared as numerous diffusely distributed fine dots along the surface of motor neurons and their proximal dendrites, and delineated their polygonal contours (Fig. 7A,B). However, after ALG+GFs treatment, the density of SYN+ vesicles around remaining CHAT+ motor neurons of the anterior horns strikingly increased when compared to all experimental groups (Fig. 7C,D). The immunoreactive profiles appeared as coarse granules of different size that were also distributed on motor neuron surface. Quantitative analysis of SYN+ vesicle expressed as % of SYN+ positive vesicles within identical fields of anterior horns in all experimental groups confirmed significant increase in ALG+GFs treated group, particularly caudally to the epicentre of injury (Fig. 7D). Interestingly, we did not see any differences in the density of SYN+ positive vesicles within segments above the lesion site (data not shown).

### CGRP positive fibres

**Figure 8:**
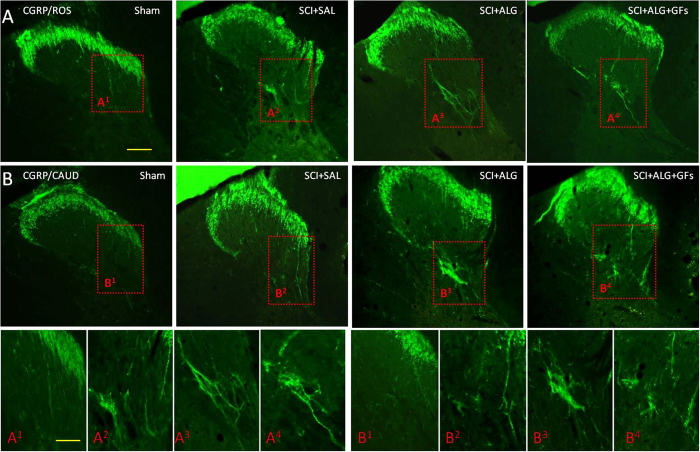
CGRP expression in transverse sections of dorsal horn/thoracic spinal cords from Sham, SCI+SAL, SCI+ALG and SCI+ALG+GFs groups 49D post-injury. Representative pictures demonstrate differences in CGRP expression, particularly in number and length of CGRP positive fibers among the experimental groups. Note, enhanced growth and branching of CGRP fibers from dorsal horn to Laminae III–V and VII in SCI+ALG and SCI+ALG+GFs groups (A^3^, B^3^, A^4^, B^4^) when compared to sham and saline rats (A^1^, B^1^, A^2^, B^2^). Scale bar = 500 μm. Lower panel shows higher magnification from corresponding regions. Scale bar = 250 μm.

**Figure 9:**
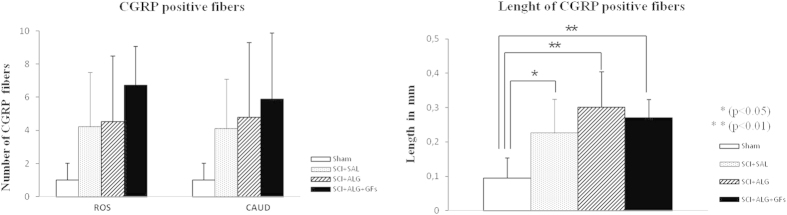
Number and length of CGRP positive fibers. (**A**) Quantitative analyses of CGRP positive fibers number showed no significant differences between individual experimental groups. (**B**) Statistical significance was observed only in the length of CGRP positive fibers between Sham and other experimental groups (SCI+SAL, SCI+ALG, SCI+ALG+GFs) (*P < 0.05, **P < 0.01, ***P < 0.001).

CGRP immunoreactivity was observed in all experimental groups (SCI+ALG+GFs, SCI+ALG, SCI+SAL) in fibres and punctuate terminals of superficial dorsal horn (Laminae I–III) and LT (LT-Lissauer’s tract) area^44^ located along the lateral edge of the dorsal horn and medial grey mater (Fig. 8). Moreover, depending on the experimental group, few individual CGRP positive fibres extending from Lamina III toward Laminae V (0.226 ± 0.099 mm/SCI+SAL) and VII (Figs 8 and 9) were detected. The longest CGRP+ fibres with the average of length 0.301 ± 0.103 mm were observed after administration of alginate biomaterial alone and alginate biomaterial with affinity-bound GFs to the injured spinal cord (0.301 ± 0.103 mm/SCI+ALG; 0.27 ± 0.053 mm/SCI+ALG+GFs). Sham spinal cord didn’t contain CGRP positive fibres extended into the intermedia spinal cord layers; however CGRP terminals within superficial dorsal horn were frequently observed. The differences associated with length of fibres show statistical significance (**P < 0.01, *P < 0.05) only between Sham and other experimental groups (SCI+SAL, SCI+ALG, SCI+ALG+GFs) (Fig. 9).

The number of immunolabeled CGRP fibres varied among individual experimental groups and areas of spinal cord. The most numerous CGRP positive fibres, forming bundles –like structures were observed in rostral segments from the lesion site after the delivery of alginate biomaterial with the affinity-bound GFs (6.7 ± 2.3/SCI+ALG+GFs, 4.5 ± 4/SCI+ALG, 4.2 ± 3.3/SCI+SAL, 1 ± 1/Sham). Average number of positive fibres was slightly decreased caudally to the epicentre of injury (5.9 ± 4/SCI+ALG+GFs, 4.8 ± 4.5/SCI+ALG, 4.1 ± 3/SCI+SAL, 1 ± 1/Sham) (Figs 8 and 9). Among the individual experimental groups in both studied parameters we did not observe statistical differences (Fig. 9).

### Axonal sprouting via BDA tracing

**Figure 10:**
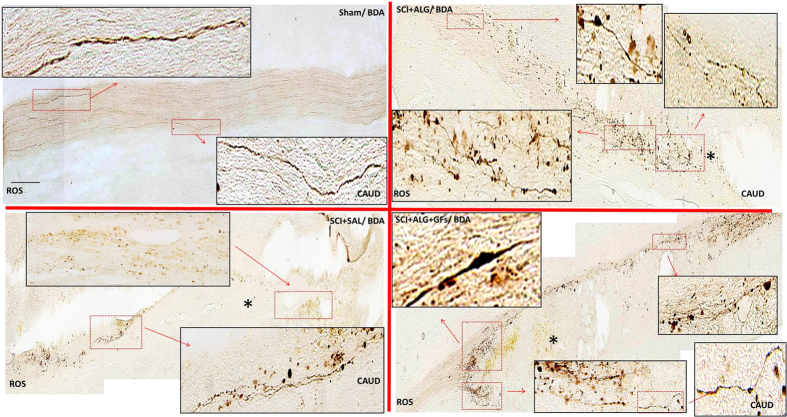
BDA labeling in the representative sagittal sections in Sham, SCI+SAL, SCI+ALG and SCI+ALG+GFs groups 49D post injury. Representative pictures and corresponding details (boxed areas) illustrate growth potential of CST fibers after SCI and individual treatments. Increased positivity of BDA was seen after alginate administration (SCI+ALG and SCI+ALG+GFs groups) where CST axons were growing around the lesion site towards disconnected caudal segment. Scale bar = 500 μm.

**Figure 11:**
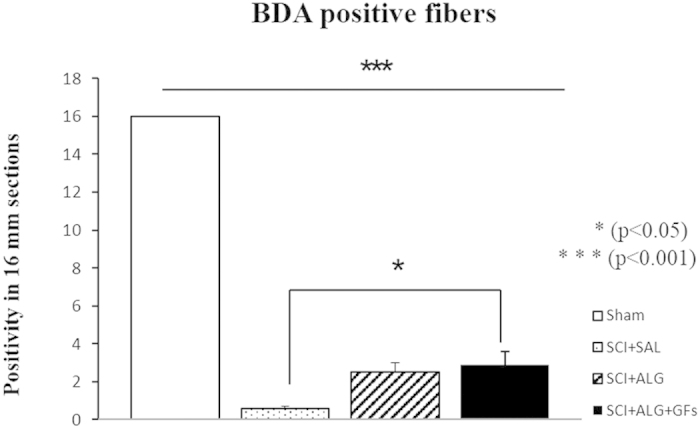
Quantification of BDA positivity in sagittal section of spinal cord. Administration of alginate enriched with growth factors leads to the significant (*P < 0.05) increase of BDA labeled fibers when compared to saline treatment.

BDA delivery to the sensorimotor cortex served to label descending CST axons of spinal cord. In sham animals, BDA-labelled CST axons were detected along the entire length of sagittal sections (16 mm) of the spinal cord; more specifically in the ventral part of the dorsal column, where stripe of organized BDA positive axons occurred (16 mm ± 0) (Figs 10 and 11). After spinal cord injury, CST axons appeared disorganized, ended above the lesion site and many cut BDA axons formed terminal structures like buttons. Re-growth of CST fibres into denervated areas of spinal cord was monitored following alginate administration. Moreover, the alginate biomaterial alone and with affinity-bound EGF/bFGF promoted increased re-growth of few BDA positive fibres through the central lesion with occasional innervations below the lesion site (2.9 mm ± 0.7 from a total 16 mm length of section) compared to saline treatment (0.6 mm ± 0.1) (Figs 10 and 11) (*P < 0.05).

### Iba1 immunohistochemistry

**Figure 12:**
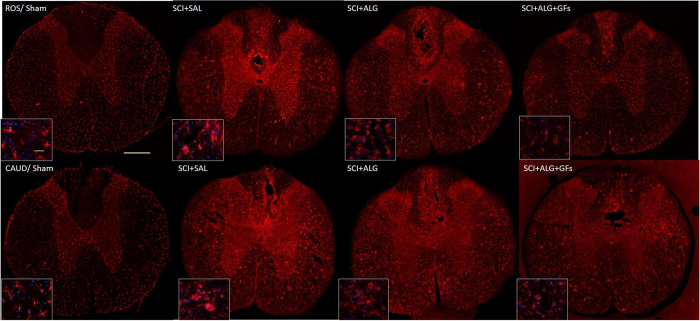
Representative transverse sections of Iba1 expression from caudal and rostral segments of spinal cord. Sections illustrate changes in activation and cell morphology after saline and alginate treatment. Baseline expression of non-active microglia in gray and white matter was observed in Sham animals. Strong activation of microglia characterized by enlarged round-shaped perikaryon with truncated, dick and ramified processes was detected in SCI+SAL group. Alginate treatment (SCI+ALG and SCI+ALG+GFs groups) inhibited activation of Iba1 positive cells. Scale bars = 500 μm, 10 μm (higher magnification of boxed area).

In order to monitor the immune response of host tissue, particularly the presence of microglia cells after alginate biomaterial treatment, Iba1 immunoreactivity in lesion site and also in the adjacent segments located 0.8 cm rostrally/caudally to the epicenter, were used. Enhancement of the microglia responsiveness and subsequent density was observed mainly after injury with injection of saline (SCI+SAL: Ros/17 ± 3, Ros-Centre/23.3 ± 3.4, Centre-Caud/22.3 ± 4.4, Caud/21.3 ± 4.9), while the decreased tendency of Iba1 expression was seen after treatment with alginate biomaterial (SCI+ALG+GFs: Ros/14.8 ± 2.1, Ros-Centre/17.3 ± 4.3, Centre-Caud/18.1 ± 3, Caud/17.4 ± 2.9; SCI+ALG: Ros/16.4 ± 2.5, Ros-Centre/20.5 ± 3.2, Centre-Caud/17.6 ± 4.2, Caud/14.2 ± 4.3) (Fig. 12, Supplementary Figure 5). Positivity of Iba1 expression in Sham animals revealed basaline levels (Ros/9.4 ± 1.5, Ros-Centre/9.3 ± 0.8, Centre-Caud/9.3 ± 1.6, Caud/9.8 ± 1.6). Among experimental groups and individual parts of spinal cord significant differences were detected (***P < 0.001, **P < 0.01, *P < 0.05, Supplementary Figure 5).

### Glial scar modulation (GFAP immunoreactivity)

**Figure 13:**
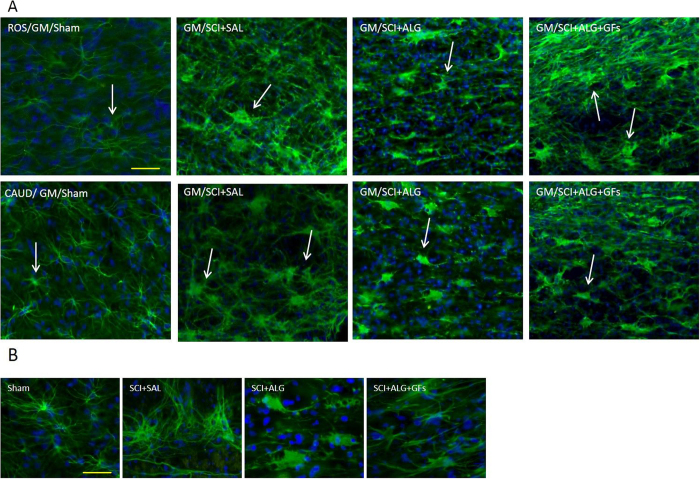
Baseline expression of GFAP-positive astrocytes with the characteristic round nuclei and slender, long processes distributed throughout both white and gray matter was revealed in Sham animals 49D post-injury. SCI and vehicle/saline treatment induced increase in GFAP immunohistochemical staining associated with cellular transformation; swollen hypertrophic appearance and short, dick processes indicating activated phenotype. Similarly alginate slightly increased the GFAP density but positive cells were without hypertrophic appearance. Scale bars = 100 μm (**A**), 50 μm (**B**).

Baseline expression of GFAP-positive astrocytes with the characteristic round small soma and slender, long processes were seen in Sham spinal cord distributed throughout white and grey matter (Ros/11 ± 1.8, Ros-Centre/10.81 ± 1.41, Centre-Caud/10.81 ± 1.41, Caud/12.6 ± 1.6) (Fig. 13). The significant response of astrocytes that resulted in increased density and change of cellular morphology was observed following SCI and saline delivery (***P < 0.001, **P < 0.01, *P < 0.05). Astrocytes assumed increased GFAP staining with subsequent cellular transformation into swollen hypertrophic appearance and short, thick processes indicating activated phenotype (Fig. 13). Similarly, alginate biomaterial treatment alone or with affinity-bound bFGF/EGF induced appearance of activated astrocytes, but with poorer ramification as seen after saline delivery (Fig. 13). The densitometry analysis revealed differences between the individual parts of 1.6 cm sections (Ros, Ros/Central, Central/Caudal, Caudal) of spinal cord. The highest positivity of GFAP was measured within Centro-Caudal site in all experimental groups (SCI+ALG+GFs: Ros/11 ± 1.8, Ros-Centre/10.81 ± 1.41, Centre-caud/10.81 ± 1.41, Caud/12.6 ± 1.6; SCI+ALG: Ros/14.9 ± 4.1, Ros-Centre/13.4 ± 3.6, Centre-Caud/16 ± 3.5, Caud/13.8 ± 3.1; SCI+SAL: Ros/17.9 ± 2.2, Ros-Centre/18 ± 3.7, Centre-Caud/21.5 ± 3.9, Caud/21.4 ± 1.6) (Supplementary Figure 6). The quantification of GFAP immunoreactivity on transverse sections from rostral and caudal segments of spinal cord also confirmed an increase in immunoreactivity caudally to the lesion site especially after saline delivery (Rostrally: 14.8 ± 4.2/SCI+ALG+GFs, 14.9 ± 4.9/SCI+ALG,18.11 ± 1.6/SCI+SAL, 13.6 ± 1.6/Sham; Caudally: 16.3 ± 3/SCI+ALG+GFs, 15.3 ± 3.4/SCI+ALG, 17.6 ± 2.4/SCI+SAL, 13.2 ± 0.5/Sham). Differences in GFAP density between experimental groups in rostro-caudal segments were without observed significant differences (Supplementary Figure 6).

### Angiogenesis

For visualization of the vascular structures, the endothelial cell marker von Willebrand Factor (vWF) was used (Supplementary Figure 7). Numerous positive blood vessels were observed in the Sham group, mainly in the white matter compared to the injured spinal cord groups (SCI+SAL or SCI+ALG), where the density of blood vessels decreased in close vicinity of the lesion site (Supplementary Figure 7). Treatment with alginate biomaterial with the affinity-bound GFs resulted in an increase of vWF positive blood vessels in the white matter and in grey matter as well, at lesion site. Results obtained from immunohistochemical analyses suggest that GFs-enriched alginate biomaterial created a suitable environment for blood vessels survival or reconstruction, but without significant differences between treatment groups (data not shown).

## Discussion

Currently, the field of SCI neurotherapeutics is still in its infancy and there are no effective ad approved therapies for SCI in humans. A contributing factor for such failed neuroregenerative processes has been attributed partly to the development of the optimal regeneration-supportive microenvironment that can initiate a neurobridge connecting disconnected spinal cord segments.

The present study clearly demonstrates that the local delivery of injectable alginate biomaterial capable of increasing the bioavailability of key growth factors such as bFGF and EGF and their appropriate presentation improve the repair of SCI through multiple mechanisms, such as: i) reducing the central lesion cavity, ii) increasing the number of surviving neurons including ChAT+ motor neurons and their synaptic connections, iii) enhancing outgrowth of CST axons, iv) preserving or stimulating formation of new blood vessels, and v) attenuating inflammation; which altogether enhance the functional recovery after SCI without sensory impairments.

Here we applied a well-characterized compression model of spinal cord injury leading to overall impairment of motor and sensory functions associated with loss of corresponding neuronal pools, overreaction of microglia/astrocytes and inability of axonal regrowth through the lesion site^45^. Using a tissue micro-proteomic approach, we established that in the time period after lesion, the nature of proteins varied throughout the spinal rostro-caudal axis. Particularly, the proteins found in caudal and rostral segments at 7 and 10 days after SCI were different compared to 3 days post injury^40^. Previous data clearly show that three days after lesion, the factors secreted in the lesion and rostral segments are anti-inflammatory and neurotrophic, while in the caudal region a cocktail of apoptotic and neurotoxic proteins predominate^30^. The present study shows that on days 7 and 10 after SCI, in the caudal segments neurotrophic factors are overexpressed, as well as adhesion molecules and signalling proteins. In contrast, in rostral segments the proteins overexpressed are involved in metabolism at the level of the mitochondria or the cytoplasm, as well as in intracellular signalling. This clearly indicates that real differences exist between the rostral and caudal segments in terms of physiological and molecular processes, and that these differences are dynamic in time. Importantly, the results indicate that the caudal region possesses all the factors that can stimulate neurite outgrowth, but these seem to be insufficient in amount and are blocked by proteoglycans. Taking into account these *ex vivo* data, we attempted to connect the rostral and caudal segments through the lesion by constructing an alginate biomaterial bridge loaded with GFs. Thus all immunohistochemical and tracing analyses were performed along the rostro-caudal axis, to better understand and define differences in pathological or regenerative processes above and below the lesion site after biomaterial treatment.

Our strategy is in line with recent pre-clinical studies performed after incomplete/complete injuries, and attempting to reconnect links with the tissue below the injury site, either bypassing the central lesion or rebuilding tissue in a cyst mediated via the application of biomaterials^46^,^47^,^48^,^49^,^50^. The novelty of our strategy is the combination of biomaterial used as a bridge together with sustained delivery of key growth factors for SCI repair. *In vitro*, this combination was found to be effective in promoting cell retention and expansion, while also enabling neural progenitor lineage differentiation *in situ*^25^. In continuity with these findings, our *in vivo* results document significant spinal cord tissue sparing, resulting in neuronal sparing that may lead to enhanced plasticity and reorganization of preserved neuronal circuits. Furthermore, the sparing of ChAT positive motor neurons may correlate with the trend of motor function improvement observed during the whole survival period in SCI rats treated with the alginate scaffold with or without GFs, in comparison to animals treated with saline.

Physiological locomotion is governed by motoneurons that receive synaptic inputs from local interneurons, descending pathways and proprioceptive sensory neurons. The convergence of proper excitatory and inhibitory inputs on motoneurons mediated by synaptic connections is required for motor control, reflexes and tonic firing of the motoneurons. Disruption of the cellular components and/or synaptic connectivity in this spinal circuitry has been implicated in motoneuron spasticity and various motoneuron disorders^45^. For this reason in this study we followed the response of ChAT motor neuron-related synaptic vesicles in segments above and below the central lesion using synaptophysin immunohistochemistry (SYN+IR). Synaptophysin is the most abundant integral membrane protein of synaptic vesicles^51^ and can be used as a marker protein of synaptic vesicles in the central and peripheral nervous systems^52^. The present data document that SYN+IR around motor neurons in the anterior horns showed similar patterns in most experimental groups, except for the group receiving ALG+GFs. In these rats, we observed more intense SYN+IR in the caudal compared to the rostral segments. These results may be linked with our proteomic data, confirming the higher expression of neurotrophic and synaptogenetic factors in the caudal segment, thus producing a favourable environment for synaptic rebuilding reflected by increased SYN+IR after ALG+GF delivery. Although our proteomic findings respond to 10 days survival following SCI, the higher level of synaptogenetic factors may be further potentiated with a GF-enriched environment, as most likely seen in the present study with ALG+GF delivery. The mechanisms mediating increase in SYN+IR may reflect several processes such as: i) up-regulation of synaptic functions after SCI, which is more likely related to the release of excitatory amino acids, or ii) may indicate plastic changes associated with formation of new synapses. Thus, to further understand changes in motoneuron synaptic connectivity after SCI treatment, the transporter systems such as vesicular glutamate and glycine transporters (VGluT1/VGluT2, GlyT2) need to be further studied.

Furthermore, SCI-induced secondary pathological processes also cause interruption of the CST tract^53^,^54^, leading to partial or complete impairment of descending motor pathways for skilled movements below the injury^2^,^55^. The compression model used in the present experimental study carried out at thoracic levels caused interruption of axon fibres corresponding to both hindpaws with some degree of spontaneous regeneration and behavioural improvements^56^. The behavioural outcome can be enhanced by promoting the axonal integrity and plasticity of the corticospinal tract and descending serotonergic pathways via GF delivery^57^. In according with these finding, our data confirm significant re-growth of BDA positive fibres observed after intraspinal injection of GF-enriched alginate biomaterial at the central lesion^58^. In contrast, delivery of alginate biomaterial alone did not induce the same effect as the GF-enriched biomaterial. The neuroprotective effect of biomaterial on axon regrowth has been described in many other *in vitro* and *in vivo* studies, where *in vitro* studies demonstrate that biomaterial promotes neural cell attachment and neurite outgrowth^59^,^60^,^61^ while *in vivo* studies show only partial regeneration after gel is implanted in the injured spinal cord^23^,^62^,^63^. The explanation for differences in axonal outgrowth seen between *in vitro* and *in vivo* may be given by multiple factors associated with inhibitory, immune, endocrine processes that are typical for the complex *in vivo* environment^21^,^64^,^65^. In addition, optimal regeneration of axons requires preserved vascular supply. Our data indicate that an alginate scaffold may provide an appropriate substrate also for the survival and re-growth of blood vessels. Furthermore, growth factors affinity-bound to the alginate scaffold promote the survival, proliferation and differentiation of microvascular cells^66^,^67^, which results in extensive collateral branching of damaged vessels and thickening of vessels within the lesion site.

Another important issue in damaged spinal cord pathology is the development of central sensitization, which often contributes to hyperalgesia and allodynia typically associated with inflammatory pain^68^. In the present study therefore we addressed the response of sensory fibres expressing calcitonin gene-related peptide (CGRP) following treatment with alginate biomaterial. Our data show that significant increase in CGRP+ fibres occurred in the dorsal horns and lateral grey matter after ALG+GF delivery. These most likely represent unmyelinated pelvic afferent fibres that convey thermal and nociceptive information^69^. Plastic re-organization of spinal neural circuitry and morphological changes in the spinal reflex pathway (primary afferent fibres and spinal interneurons) may be responsible for serious post-injury complications that could lead to uncontrolled excitatory activity of glutamate-driven sympathetic preganglionic neurons, and similarly to loss of inhibitory GABAergic/glycinergic interneurons that could have an impact on increased bilateral hind limb sensitivity to cold. Although the administration of GF-enriched alginate biomaterial promoted extending of CGRP positive fibres, we did not observe adverse sensory response to cold, such as observed after saline or pure alginate delivery. Responses of the hind limbs were relatively stable in the SCI+ALG+GF treated group during the whole survival period, with intensity similar to that in the sham controls. From our results we can speculate that alginate biomaterial with affinity-bound growth factors enhanced changes in CGRP fibres, but without behavioural adverse sensory response.

Central sensitization of spinal neurons or neuronal hyper-responsiveness and alterations in behavioural pain thresholds may be also in close correlation with microglial activation, as pointed out in some recent studies^70^,^71^,^72^. It is known that release of excitatory amino acids^73^, interleukin-1^74^, and prostaglandin E2^75^ by microglia actively participate in the generation of central sensitization after SCI. On the basis of this hypothesis we can conclude that significant microglia response after saline delivery could induce an increase in central sensitization of spinal neurons and promote the kind of adverse sensory response to cold detected in the present study. However, ALG-GF treatment causing attenuation of microglia may lead to normalization of sensory behaviour.

Reactive astrocytes together with microglia and meningeal fibroblasts are known to participate in scar formation, representing a mechanical and chemical barrier for nerve tissue regeneration^76^. Significant differences in GFAP+IR between the experimental groups were observed spatially, mainly in the central and caudal segments. However, treatment with ALG and ALG+GFs did not attenuate the activation and proliferation of astrocytes after SCI, which may ultimately contribute to glia scarring at the central lesion site.

In the present experimental study we tried to define the efficacy of usage of alginate itself as well alginate enriched with GFs for spinal cord repair. EGF and bFGF were selected due to their stable and high binding properties, as well as long-term sustained GF release from alginates, confirmed *in vitro*^25^. Although these factors are important for their mitotic and partially differential properties for endogenous neural progenitors and their ability to accelerate neovascularisation, they may also contribute to astrogliosis and tissue scarring. In future experiments therefore, other neurotrophins such as BDNF, GDNF, NT-3 with neuroprotective action, or VEGF, PDGF with angiogenic properties should be considered for incorporation into one alginate device. In addition, simultaneous digestion of Chondroitin Sulfate Proteoglycans by chondroitinase ABC (ChABC) should be considered as well^8^,^9^,^43^. From the surgical point of view, the alginate scaffold described herein was injected into irregular spinal cord cavities, where it adjusted itself into the cavity shape and in the presence of calcium ions could undergo gelation *in situ*. This type of non-invasive technique for vehicle administration is potentially advantageous in particular when considering the fragility of the spinal cord site.

## Conclusions

We describe here that an affinity-binding alginate scaffold with sustained presentation of bFGF/EGF has the potential to serve as a useful delivery vehicle in a certain model of SCI damage, with proven capability to promote neuronal sparing, modulate motoneuron synapses, enhance regrowth of BDA-positive CST fibres and the presence of vWF positive vessels at the injury site, as well as enhancing changes in CGRP fibres, but without behavioural adverse sensory response.

Furthermore, combinatory-based local therapy using a biomaterial scaffold with neurotrophic factors, inhibitory molecules, enzymes that could digest deposits of extracellular matrix, e.g. chondroitin sulphate proteoglycans, as well as stem cells, may provide more advanced therapy for spinal cord repair.

## Additional Information

**How to cite this article**: Grulova, I. *et al.* Delivery of Alginate Scaffold Releasing Two Trophic Factors for Spinal Cord Injury Repair. *Sci. Rep.* **5**, 13702; doi: 10.1038/srep13702 (2015).

## Supplementary Material

Supplementary Information

## Figures and Tables

**Table 1:** Specific proteins present in tissue in lesion, rostral at 7days, rostral at 10 days, caudal at 7 days or in caudal at 10days.

Number Accession	Protein Name	Lesion	Rostral 7D	Rostral 10D	Caudal 7D	Caudal 10D
P11505-5	Plasma membrane 1, isoform CRA_b	X				
D4A5×7	Ganglioside-induced differentiation-associated-protein 1	X				
F1LMW7	Myristoylated alanine-rich C-kinase substrate	X				
F1LXA0	NADH dehydrogenase 1 alpha subcomplex	X				
Q64591	2,4-dienoyl-CoA reductase, CRA_a	X				
M0R4S2	Apolipoprotein D	X				
M0R655	Protein Fmnl2	X				
P04904	Glutathione S-transferase alpha-3 protein	X				
P31647	Sodium- and chloride-dependent GABA transporter 3	X				
P35213	14-3-3 protein beta/alpha	X				
P62747	Rho-related GTP-binding protein RhoB	X				
P97532	3-mercaptopyruvate sulfurtransferase	X				
Q02563	Synapticvesicleglycoprotein 2A	X				
Q04462	Valine-tRNA ligase	X				
B0BNM9	Glycolipidtransferprotein		X			
D3ZDK7	ProteinPgp		X			
M0R5K9	40S ribosomal protein S18		X			
D4ACB8	Chaperonin subunit 8, isoform CRA_a		X			
O35814	Stress-induced-phosphoprotein 1		X			
P18418	Calreticulin		X			
Q6PDW4	Proteasomesubunit beta type-1		X			
Q68G41	Dodecenoyl-Coenzyme A delta isomerase		X			
P31399	ATP synthase subunit d, mitochondrial		X			
P39069	Adenylate kinase isoenzyme 1		X			
Q4KM73	UMP-CMP kinase		X			
Q5U300	Ubiquitin-like modifier-activatingenzy. 1		X			
Q5XIN6	LETM1 and EF-hand domain protein 1		X			
Q920L2	Succinatedehydrogenaseflavoprotein		X			
Q9WVB1	Ras-relatedprotein Rab-6A		X			
B0BNM9	Glycolipidtransferprotein		X			
F1LM47	Protein Sucla2			X		
O35244	Peroxiredoxin-6			X		
P07722	Myelin-associatedglycoprotein			X		
P32089	Tricarboxylate transport protein			X		
P51583	Multifunctionalprotein ADE2			X		
Q66H18	Protein Sypl1			X		
Q68FQ0	T-complexprotein 1 subunit epsilon			X		
Q9QUL6	Vesicle-fusing ATPase			X		
Q9QX69	LanC-likeprotein 1			X		
Q9QZR6	Septin-9			X		
F1LM47	Protein Sucla2			X		
B0BNK1	Protein Rab5c				X	
P47942	Dihydropyrimidinaserelatedprotein2				X	
B2GUZ5	F-actin-capping protein subunit alpha-1				X	
D3ZNW5	Neurofascin				X	
P62986	Ubiquitin-60S ribosomal protein L40				X	
G3V9L3	MAGUK p55 subfamily member 3				X	
P19234	NADH dehydrogenaseflavoprotein 2				X	
D4A8U7	Dynactinsubunit 1					X
E2RUH2	Ribonucleaseinhibitor					X
E9PT65	ProteinRdx					X
G3V8A5	Vacuolar protein sorting protein 35					X
G3V8C4	Chloride intracellular channel protein 4					X
Q63228	Glia maturation factor beta					X
P18484	AP-2 complexsubunit alpha-2					X
P97852	Peroxisomalmultifunctional enzyme type 2					X
Q5I0D7	Xaa-Pro dipeptidase					X
Q7TMC7	Ab2-417					X
Q8VBU2.	NDRG2					X
