# Supplementary material for: Delivery of Alginate Scaffold Releasing Two Trophic Factors for Spinal Cord Injury Repair
Source: Sci Rep. 2015 Sep 8;5:13702. doi: 10.1038/srep13702 (PMC4562265; doi:10.1038/srep13702)

Delivery of Alginate Scaffold Releasing Two Trophic Factors for Spinal Cord Injury Repair

Grulova I.[1], Slovinska L.[1], Blaško J.[1], Devaux S.[1,2], Wisztorski M.[2], Salzet M.[2], Fournier I.[2], Kryukov O.[3], Cohen S.[3], Cizkova D.[1,2*]

[1]Institute of Neurobiology, Center of Excellence for Brain Research, Department of Regenerative Medicine and Stem Cell Therapy, Slovak Academy of Sciences, Soltesovej 4-6, 040 01 Kosice, Slovakia

[2]Laboratoire PRISM : Protéomique, Réponse Inflammatoire, Spectrométrie de Masse, INSERM U1192, Bât SN3, 1er étage, Université de Lille 1, F-59655 Villeneuve d'Ascq, France

[3]The Center of Regenerative Medicine and Stem Cell Research and The Avram and Stella Goldstein-Goren Department of Biotechnology Engineering, Ben-Gurion University of the Negev, Beer Sheva, Israel

*** Corresponding author:**

D.V.M. Dasa Cizkova, PhD, DrSc.

Institute of Neurobiology, Slovak Academy of Sciences,

Center of Excellence for Brain Research,

Department of Regenerative Medicine and Stem Cell Therapy

Soltesovej 4-6

04001 Kosice,

Slovakia

Phone: +421 55 678 5064, Fax: +421 55 678 5074

Email: cizkova.dasa@gmail.com

Keywords: spinal cord injury; alginate; regeneration; therapeutic approach, tissue microproteomic, microglia

Supplementary information

Supplementary Figure 1. Illustration of the bregma and lambda anatomical location**. B** Note four openings for BDA administration targeting the sensorimotor cortex.

Supplementary Figure 2. Schematic drawing of individual experimental steps of present study.

Supplementary Figure 3. Concept of dissected spinal cord segments consisting of rostral (Th5-7), central (Th8-9), caudal (Th10-12) segments processed for immunohistochemical analyses.

Supplementary Figure 4. Schematic illustration of areas for cells quantification. **A** Quantification of NeuN was performed bilaterally in 3 squares (200 x 200 μm) in the Laminae I-IV (dorsal horn) (1), Laminae IV-V (intermediate zone) (2) and Laminae VIII-IX (ventral horn) (3). **B** Evaluation of Iba1 positivity was performed bilaterally within 3 squares (150 x 150 μm) in grey matter- Lamina VII (1) and white matter – lateral (2) as well as ventral funiculi (3). Scale bars: 500 μm, 10 μm.

Supplementary Figure 5. Densitometric analyses of Iba1 positivity through the 1.6 cm sagittal sections of spinal cord. Sham tissue exhibits limited levels of Iba1 expression and low background staining. The highest expression of Iba1 positivity was detected following injury and saline treatment. Intraspinal administration of pure and enriched alginate showed significant differences in Iba1 expression between experimental groups mainly caudally from the lesion site (*** $P < 0.001$, ** $P < 0.01$, * $P <0.05$).

Supplementary Figure 6. Densitometric analyses of GFAP positivity in Sham, SCI+SAL, SCI+ALG and SCI+ALG+GFs groups 49D post-injury. **A** Significant differences among experimental groups and individual parts of spinal cord was detected in 1.6 cm sagittal sections from central lesion (***$P < 0.001$, **$P < 0.01$, *$P < 0.05$). **B** GFAP expression on transverse sections from rostral and caudal segments (0.8 cm from the lesion site) was without visible significance.

Supplementary Figure 7. Effect of SCI and treatment on spontaneous recovery of vWF positive blood vessels. The longitudinal sections of injured spinal cord showed formation of

new blood vessels in close vicinity of lesion and also in lesion site after alginate treatment. vWF expression was enhanced not only in the penetrating blood vessel but also in cell bodies found close to the injury site, but mainly in rostral direction. The sham tissue exhibits homogeneous distribution of vWF staining throughout the whole section with visible structure of vessels in white matter. Scale bar: 500 μm.

Supplementary Figure 1

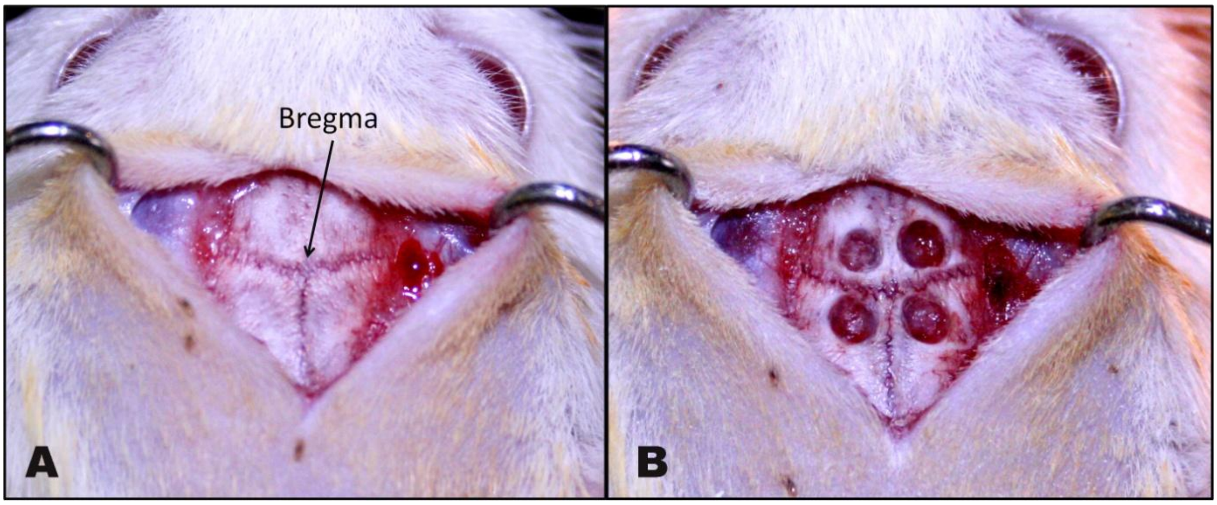

Supplementary Figure 2

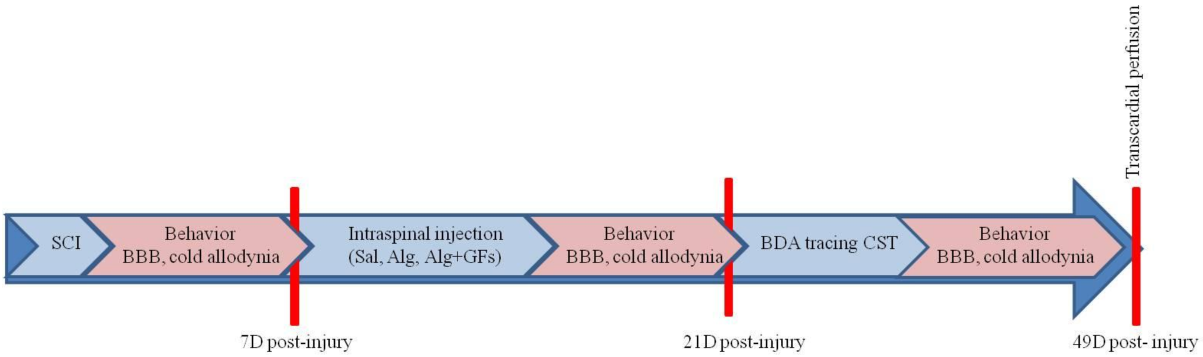

Supplementary Figure 3

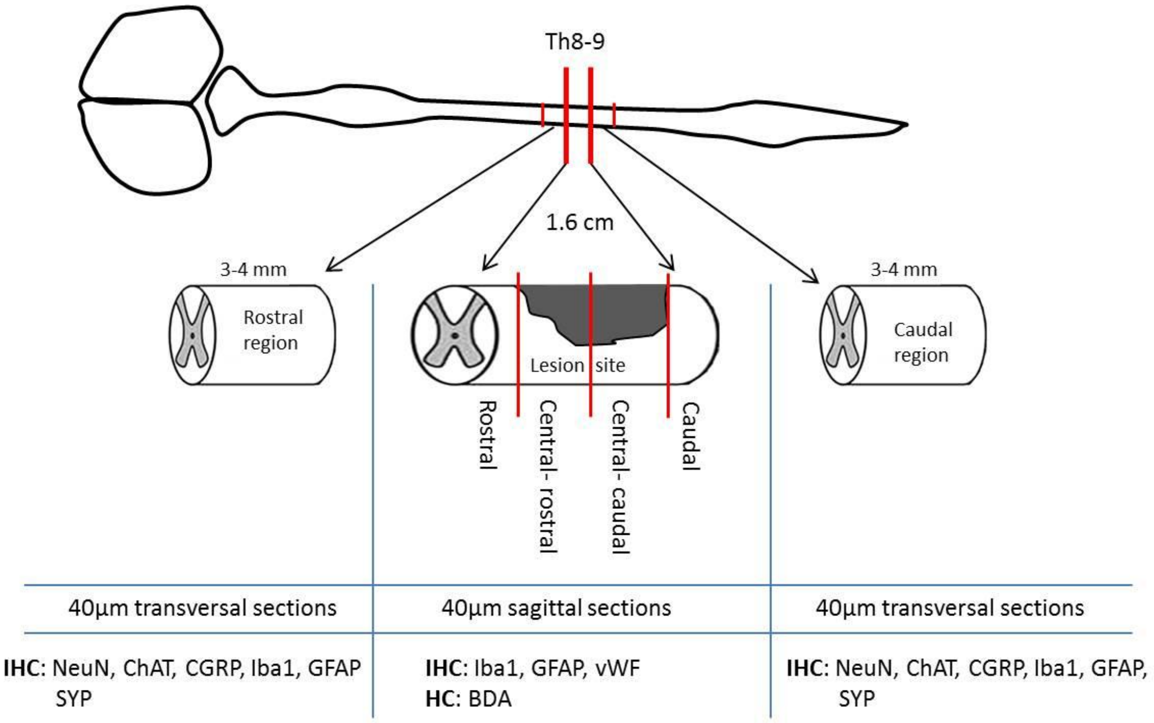

Supplementary Figure 4

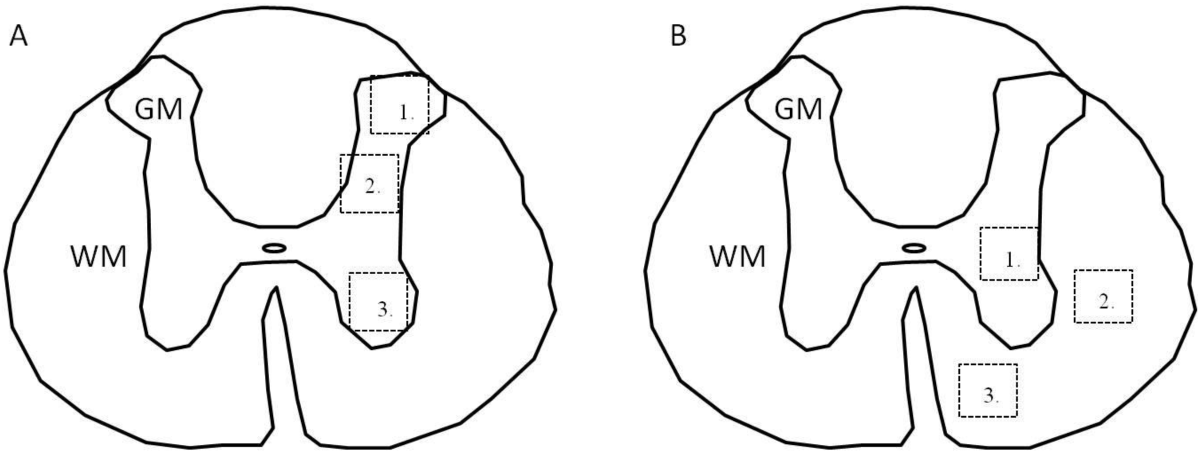

Supplementary Figure 5

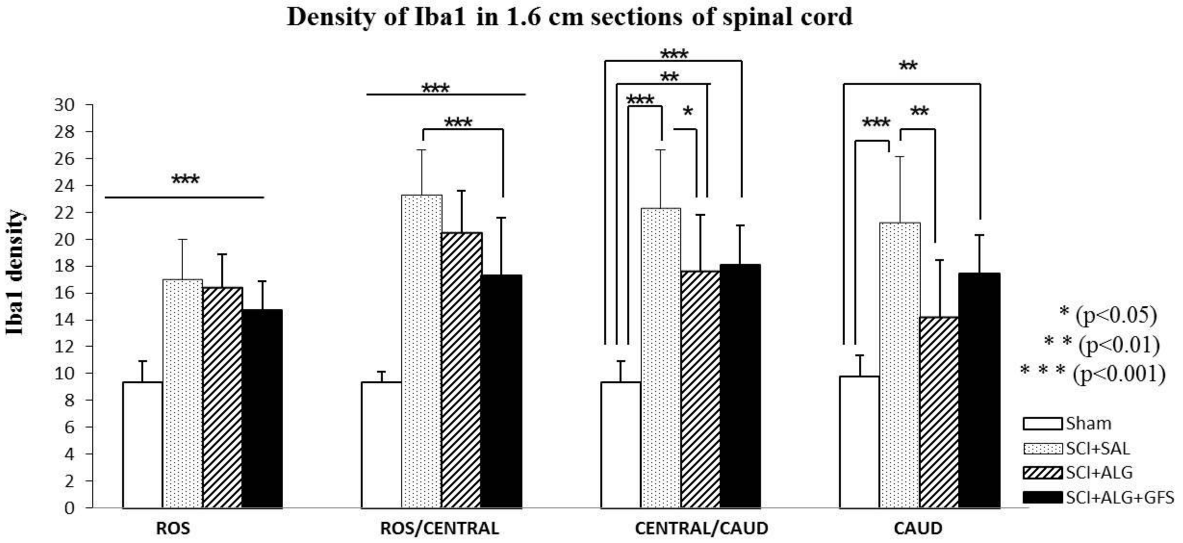

Supplementary Figure 6

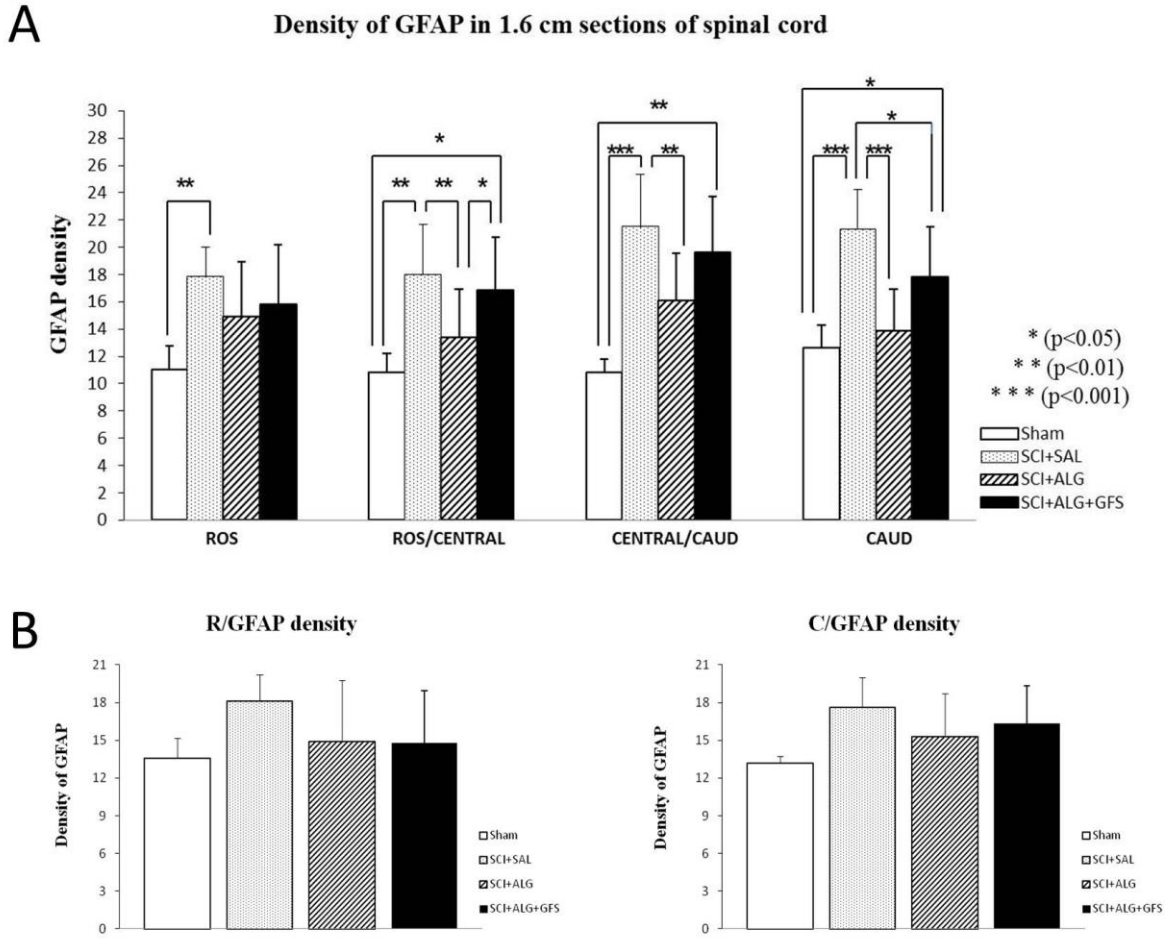

Supplementary Figure 7

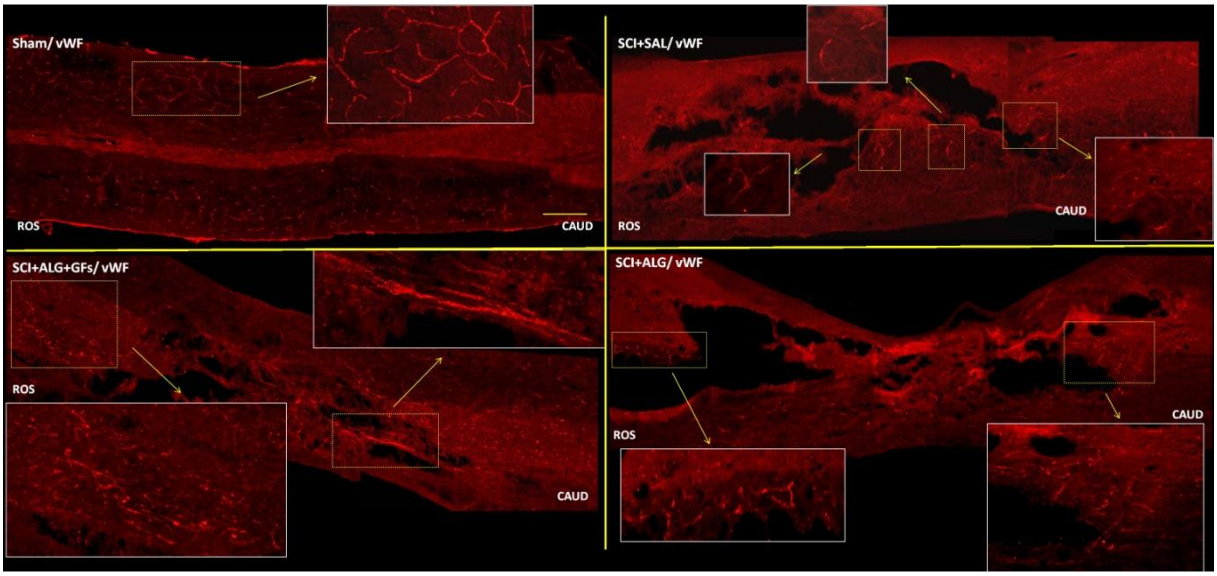

Supplement: Supplementary Information [file srep13702-s1.pdf]
